# Human neuron activity during an 83-minute movie from 2,286 neurons and 29 patients

**DOI:** 10.1038/s41597-026-07955-0

**Published:** 2026-07-30

**Authors:** Alana Darcher, Franziska Gerken, Johannes Niediek, Marcel S. Kehl, Thomas P. Reber, Stefanie Liebe, Laura Nett, Attila Racz, Lukas Kunz, Bernhard Staresina, Rachel Rapp, Pedro J. Gonçalves, Ismail Elezi, Valeri Borger, Rainer Surges, Laura Leal-Taixé, Jakob H. Macke, Florian Mormann

**Affiliations:** 1 Department of Epileptology, University Hospital Bonn, Bonn, Germany; 2 Dynamic Vision and Learning Group, Technical University of Munich, Munich, Germany; 3 Machine Learning Group, Technische Universität Berlin, Berlin, Germany; 4 Department of Experimental Psychology, University of Oxford, Oxford, UK; 5 Faculty of Psychology, UniDistance Suisse, Brig, Switzerland; 6 Department of Epileptology and Neurology, University Hospital Tübingen, Tübingen, Germany; 7 Hertie Institute for Clinical Brain Science, University of Tübingen, Tübingen, Germany; 8 Hertie Institute for Artificial Intelligence for Brain Health, University of Tübingen, Tübingen, Germany; 9 Oxford Centre for Human Brain Activity, Centre for Integrative Neuroimaging, Department of Psychiatry, University of Oxford, Oxford, UK; 10 Machine Learning in Science, Excellence Cluster Machine Learning and Tübingen AI Center, University of Tübingen, Tübingen, Germany; 11 VIB Center for AI and Computational Biology, VIB, Leuven, Belgium; 12 VIB Center for Neuroscience Leuven, VIB, Leuven, Belgium; 13 Departments of Computer Science and Electrical Engineering, KU Leuven, Leuven, Belgium; 14 Department of Neurosurgery, University Hospital Bonn, Bonn, Germany; 15 Empirical Inference, Max Planck Institute for Intelligent Systems, Tübingen, Germany; 16 Present Address: Huawei, London, United Kingdom; 17 Present Address: NVIDIA Srl Italy, Milan, Italy; 18 Present Address: ELLIS Institute Tübingen, Tübingen, Germany

## Abstract

Despite a growing trend towards more naturalistic experiments, few single-unit datasets collected during dynamic and naturalistic stimuli have been released, and none using a full-length movie. Here, we present SUMMER (Single Unit activity during a Movie in the human Medial temporal lobe via Electrophysiological Recordings), a dataset containing recordings from 2,286 neurons from the human amygdala, hippocampus, entorhinal cortex, parahippocampal cortex, and neighboring structures during the complete presentation of the commercial film *500 Days of Summer* to 29 intracranially implanted patients. We provide a rich set of frame-wise annotations spanning the entirety of the movie’s 83-minute runtime, which systematically label the most salient narrative and visual elements, from characters and locations to camera cuts and main character speech. This Neurodata Without Borders-formatted dataset contains the spike times and corresponding mean waveforms from all recorded neurons, along with demographic information and electrode localizations. For technical validation, we provide spike-sorting metrics and demonstrate the tuning of individual neurons to movie features. We additionally provide decoding results from a machine learning-based pipeline for predicting movie features from neuronal population activity. To facilitate immediate use, we offer the dataset in a machine learning-ready format alongside a codebase demonstrating both neural feature and movie feature prediction tasks. This dataset offers a valuable foundation for exploring how the human brain processes semantic content, particularly in real-world contexts involving dynamic and naturalistic stimuli.

## Background & Summary

What information is represented by neurons in the human medial temporal lobe? Previous studies of epilepsy patients with intracranially implanted electrodes reveal that neurons in the human medial temporal lobe (MTL) underlie various aspects of human memory, including semantic^1–6^, episodic^7–10^, spatial^11–13^, and working memory^14–16^, as well as sensory perception^17–22^. The experimental approaches employed in the majority of these studies have largely used trial-based paradigms with static stimuli, which offer practical advantages like structured time-alignment and isolated experimental conditions. However, it is not clear if findings from paradigms based on static stimuli translate to dynamic presentations, which are more similar to those encountered during natural vision. Recent work has, for example, shown that responses to faces by macaque inferior temporal neurons are considerably affected by the temporal continuity of the stimulus^23^. Static stimuli also preclude the investigation of action sequences and other time-evolving changes in content. Emerging evidence from studies using movies suggests that the representation of action may be more common across the human cortex than previously expected^24^. These findings demonstrate a need for more naturalistic experimental approaches^25^. Naturalistic stimuli such as movies are an intermediate stage between the laboratory and the natural world and offer the means to extend our current understanding of how various types of information are processed and represented by the brain.

**Fig. 1:**
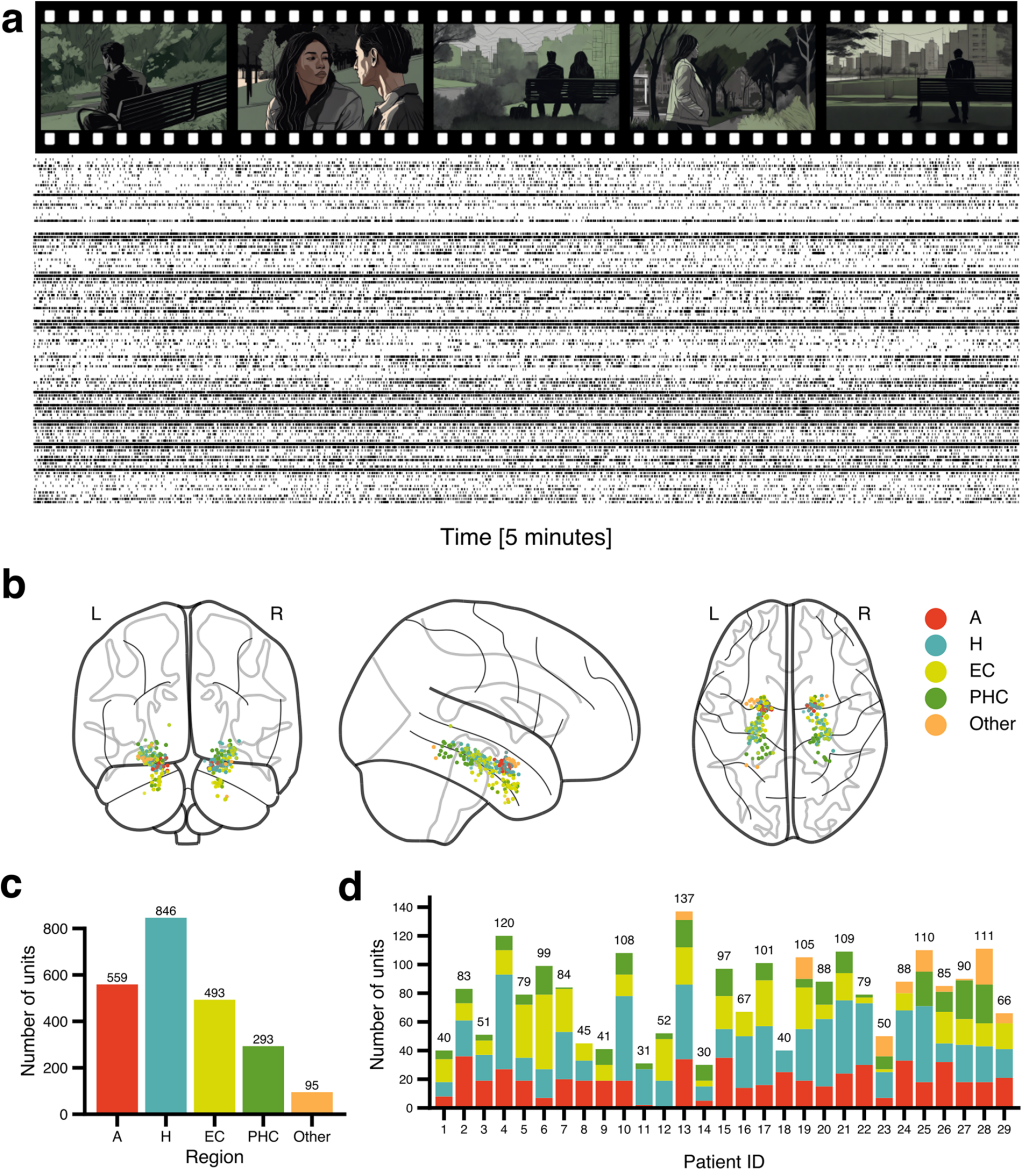
Overview of the neural data. (**a**) Five minutes of neuronal activity for the 108 neurons recorded from participant 10 during the film presentation. Each patient watched the entire 83-minute movie. Example frames were generated by stable diffusion as the original frames could not be included due to copyright. (**b**) Locations of implanted Behnke-Fried microwire bundles for all patients, shown in Montreal Neurological Institute (MNI) ICBM152 coordinates^60^. Regions: amygdala (A), hippocampus (H), entorhinal cortex (EC), parahippocampal cortex (PHC), and neighboring structures (Other). (**c**) Distribution of recorded neurons across brain regions. (**d**) Distribution of neurons across patients, stratified by brain region.

**Fig. 2:**
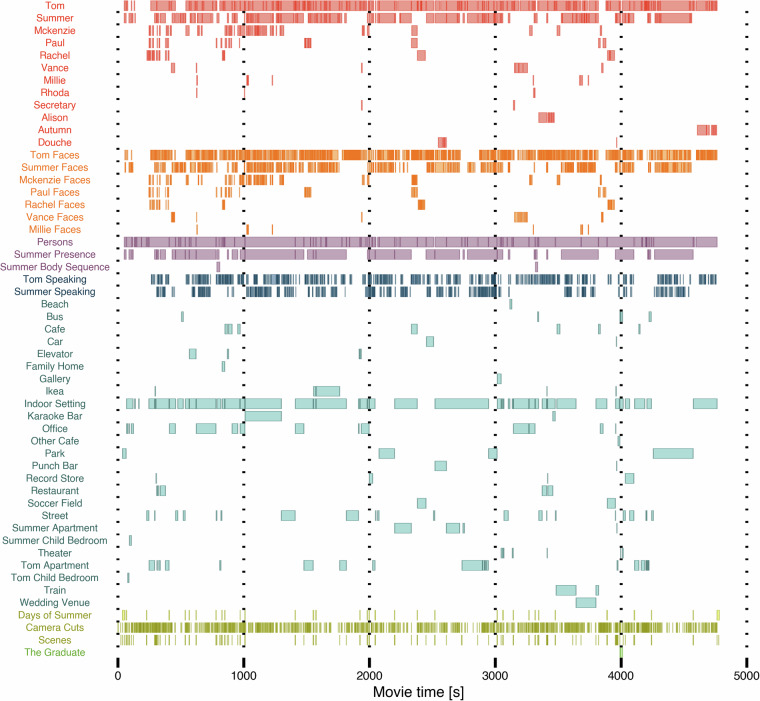
Overview of movie annotations. Presence of each annotation across the entire movie *500 Days of Summer*. Movie duration is 83 min (5029.68 s), including film credits. Label categories are characters as named in the script (red), character face appearances (orange), character-related elements (purple), speech occurrences (dark blue), locations (blue), visual transitions (spring green), and an interposed scene from the movie *The Graduate* (green).

**Fig. 3:**
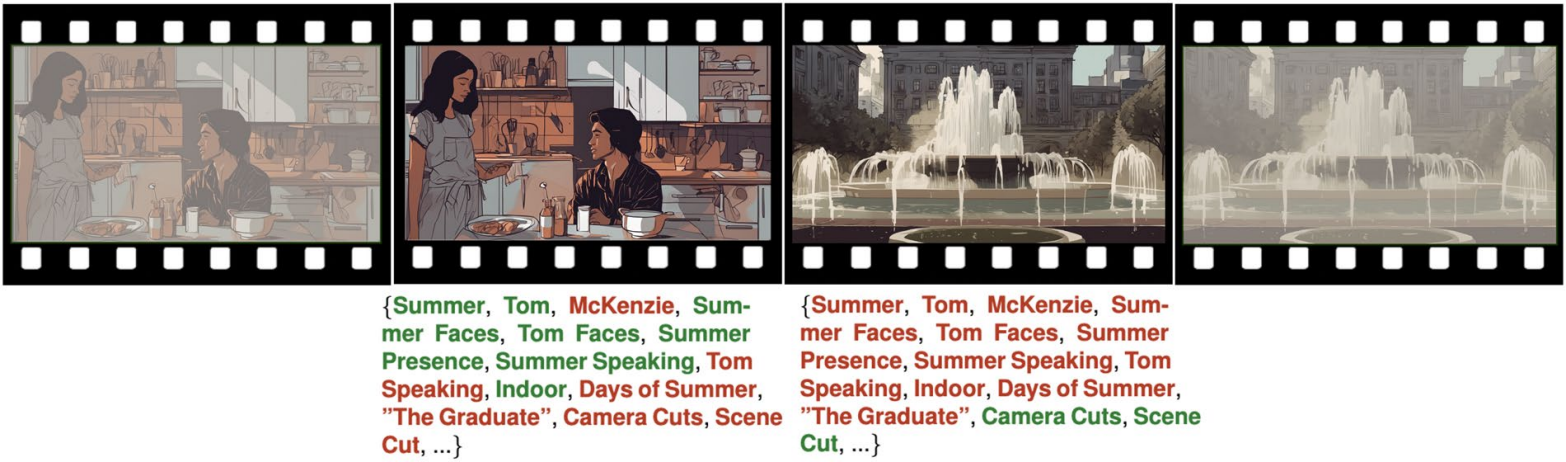
Annotations at a glance. The sequence captures a transition from an indoor kitchen scene (left two frames) to an outdoor fountain (right two frames). An example set of binary labels are given below the two inner frames (non-greyed): green indicates a present or “true” feature (e.g. Summer Presence, Indoor), while red indicates absence. The movie is labeled on a frame-by-frame basis, with Camera Cuts and Scene Cuts coded as “present” for the frame that introduces the new scene.

We present SUMMER (Single Unit activity during a Movie in the human Medial Temporal Lobe via Electrophysiological Recordings)^26^, a dataset comprising the spiking activity of 2,286 neurons recorded from the medial temporal lobes of 29 intracranially implanted patients (Table 1) during the presentation of a commercial film *500 Days of Summer* (2009, 83 minute runtime, Fig. 1a). To date, few datasets using movies as stimuli have been released^27–33^, and none of this length in humans with single-unit resolution. Alongside the spiking and waveform data, we provide a rich set of frame-by-frame annotations capturing the film’s content. These annotations cover 53 distinct features of the movie, such as character identities (including general presence and face appearance, and speech for the principal roles), locations, and visual transitions (Figs. 2, 3). Using this dataset, we previously found that populations of MTL neurons represent characters, indoor/outdoor setting, and visual transitions during dynamic presentation. Our findings showed that event boundaries are reliably represented by individual neurons, while representations of character- or setting-related information relied more strongly on population activity^34^.

**Table 1:** Patient neural data and demographics.

ID	No. electrode bundles	Regions	No. units (SU/MU)	Age	Gender
1	6	PHC, EC, AH, A	40 (25/15)	54	M
2	8	PHC, EC, AH, PH, A	83 (45/38)	32	F
3	11	PHC, EC, AH, MH, A	51 (20/31)	42	F
4	9	PHC, EC, AH, PH, A	120 (72/48)	48	M
5	10	PHC, EC, AH, PH, A	79 (37/42)	24	M
6	9	PHC, EC, AH, PH, A	99 (48/51)	26	M
7	10	PHC, EC, AH, PH, A	84 (54/30)	27	M
8	6	EC, AH, PH, A	45 (18/27)	52	F
9	3	PHC, EC, A	41 (21/20)	19	M
10	9	PHC, EC, AH, PH, A	108 (70/38)	44	F
11	6	PHC, AH, PH, A	31 (12/19)	62	M
12	8	PHC, EC, AH, PH	52 (22/30)	36	M
13	9	PHC, EC, AH, PH, A, FF	137 (86/51)	41	M
14	8	PHC, EC, AH, PH, A	30 (6/24)	26	F
15	10	PHC, EC, AH, PH, A	97 (46/51)	35	F
16	8	EC, AH, PH, A	67 (25/42)	31	F
17	9	PHC, EC, AH, PH, A	101 (43/58)	22	F
18	5	AH, PH, A	40 (18/22)	22	F
19	10	PHC, EC, AH, PH, A, LG	105 (48/57)	41	F
20	10	PHC, EC, AH, PH, A	88 (50/38)	39	F
21	9	A, PHC, EC, AH, PH	109 (55/54)	29	M
22	9	PHC, EC, AH, PH, A	79 (46/33)	41	F
23	9	PHC, EC, AH, PH, A, PIC	50 (25/25)	54	F
24	10	EC, AH, PH, A, PIC, PRC	88 (58/30)	60	F
25	9	PHC, AH, PH, A, PIC	110 (56/54)	49	M
26	10	PHC, EC, AH, PH, A, PIC	85 (41/44)	40	M
27	6	PHC, EC, AH, PH, A, PIC	90 (56/34)	25	F
28	9	PHC, EC, AH, PH, A, PIC	111 (59/52)	53	F
29	10	EC, AH, PH, A, PIC	66 (28/38)	50	F
**Total: 29**	**Total: 245, Mean: 9.97**		**Total: 2286 (1190/1096)**	**Mean: 38.76 (12.11)**	**F: 17, M: 12**

Number of electrodes includes only those from which at least one neuron was recorded. Region abbreviations: amygdala (A), anterior hippocampus (AH), middle hippocampus (MH), posterior hippocampus (PH), entorhinal cortex (EC), parahippocampal cortex (PHC), piriform cortex (PIC), fusiform gyrus (FF), lingual gyrus (LG), and perirhinal cortex (PRC).

**Fig. 4:**
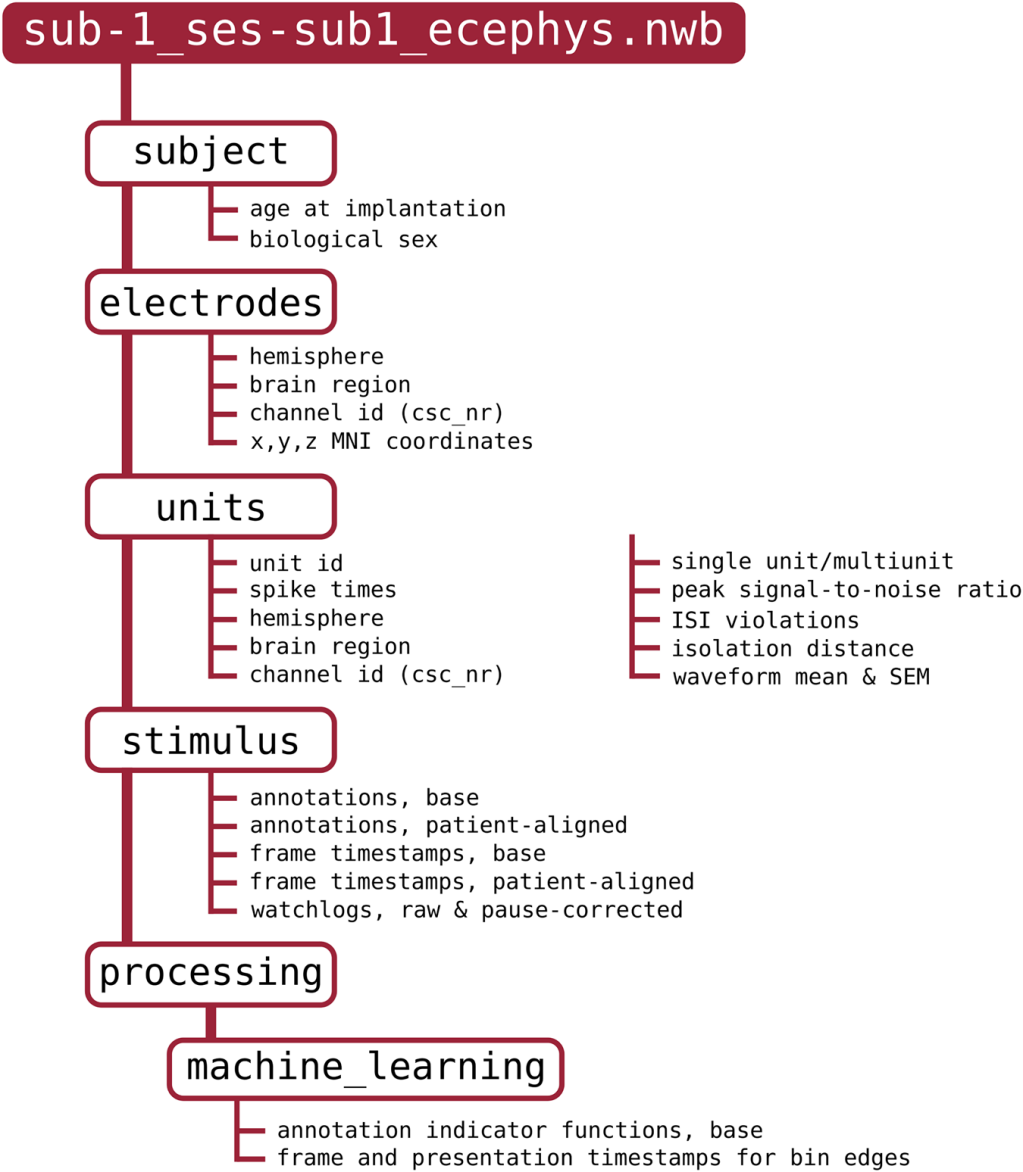
Simplified schematic of the Neurodata Without Borders file structure. Example contents of the Neurodata Without Borders (NWB) file for participant 1 (sub-1_ses-sub1_ecephys.nwb). Non-filled bubbles indicate containers for data especially relevant to analysis. For simplicity, we omit meta-data containers which are unnecessary for most analyses from the illustration. The elements attached to each container describe the contained information. csc_nr: continuously sampled channel number; MNI: Montreal Neurological Institute; SEM: standard error of the mean.

**Fig. 5:**
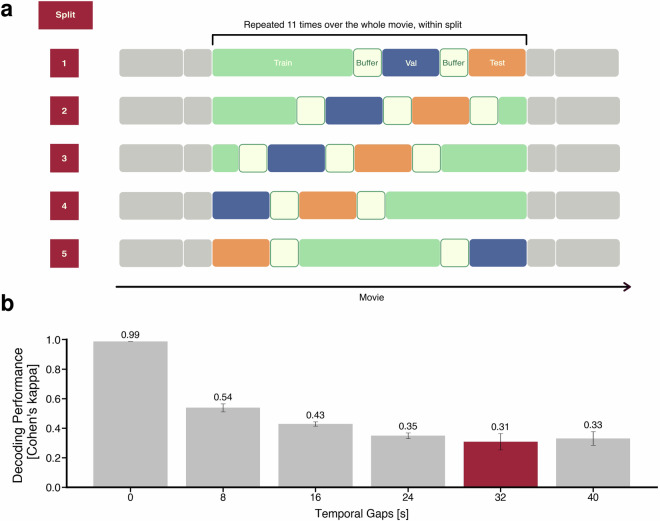
Temporal buffers prevent data leakage. (**a**) Schematic of the recommended data splits for a five-fold cross-validation setup. To account for temporal autocorrelation, the continuous movie data should be segmented into training (green), validation (blue), and test (orange) sets, separated by temporal buffers (white). This process is repeated 11 times across the duration of the movie within each split. (**b**) Decoding performance for the Summer label as a function of buffer length (temporal gaps, white cells in panel a). Small gaps result in artificially inflated performance due to autocorrelation between adjacent samples. Increasing the buffer to 32 s (red) establishes a stable, reliable baseline, demonstrating that rigorous temporal partitioning is essential to prevent over-optimistic evaluations of continuous stimuli.

All implantations and recordings in our dataset were performed at the Department of Epileptology of the University Hospital Bonn, Germany, with microwires implanted in the amygdala, hippocampus, entorhinal cortex, parahippocampal cortex, and neighboring structures as each patient watched the entire film (Fig. 1b–d). Independently, the film was also used as a stimulus in a separate functional magnetic resonance (fMRI) study by another group^27,28^. To facilitate studies of semantic, sensory, and other types of processing, we provide a comprehensive set of frame-wise movie annotations (Fig. 2), created using a combination of manual and semi-automated methods, that significantly expand upon our previous work^34^. These detailed annotations capture key narrative and visual elements (Fig. 3), including the occurrence of all named characters (12 in total), character-related elements such as face appearance and general visibility (9), the depiction of distinct locations (25), transitions between sequences and scenes (2), main character speech (2), and an interposed scene from the movie *The Graduate* (1) (Table 2). To enable broad and stable access, the dataset^26^ is provided in the Neurodata Without Borders (NWB) format (Fig. 4), supported by a codebase that streamlines data loading and integration. We include code modules that structure the NWB content for machine-learning pipelines and provide recommended data splits to avoid temporal autocorrelation (Fig. 5), lowering the barrier for users to initialize training tasks directly on the data.

**Table 2:** Overview of movie annotations.

Label	Stimulus	Category	Appearance Percentage	Onset Percentage	Method
Tom	Visual	Character	74.4097	0.1598	Manual
Summer	Visual	Character	49.5137	0.1121	Manual
McKenzie	Visual	Character	8.5015	0.0437	Manual
Rachel	Visual	Character	3.9008	0.0159	Manual
Paul	Visual	Character	3.5493	0.0223	Manual
Vance	Visual	Character	2.7707	0.0064	Manual
Autumn	Visual	Character	2.0987	0.0064	Manual
Alison	Visual	Character	1.7917	0.0056	Manual
Douche	Visual	Character	1.0179	0.0048	Manual
Millie	Visual	Character	0.8820	0.0072	Manual
Rhoda	Visual	Character	0.2990	0.0032	Manual
Secretary	Visual	Character	0.2863	0.0016	Manual
Tom Faces	Visual	Character face appearance	42.0676	1.6518	Manual
Summer Faces	Visual	Character face appearance	28.0214	0.7778	Mix
McKenzie Faces	Visual	Character face appearance	4.7351	0.1853	Mix
Paul Faces	Visual	Character face appearance	2.8518	0.0533	Mix
Rachel Faces	Visual	Character face appearance	2.5202	0.0581	Mix
Vance Faces	Visual	Character face appearance	2.1520	0.0334	Mix
Millie Faces	Visual	Character face appearance	0.3292	0.0262	Mix
Persons	Visual	Character-related	88.8574	0.0708	Manual
Summer Presence	Visual	Character-related	60.3779	0.0326	Mix
Summer Body Sequence	Visual	Character-related	0.7961	0.0016	Manual
Tom Speaking	Audio	Speech	17.6956	0.3444	Manual
Summer Speaking	Audio	Speech	11.2905	0.2513	Manual
Indoor*	Visual	Location	63.1367	0.0342	Manual
Outdoor*	Visual	Location	30.9632	0.0374	Manual
Office	Visual	Location	12.9025	0.0111	Manual
Park	Visual	Location	10.6392	0.0032	Manual
Tom Apartment	Visual	Location	10.5835	0.0167	Manual
Street	Visual	Location	8.5142	0.0167	Manual
Karaoke Bar	Visual	Location	6.1149	0.0016	Manual
Summer Apartment	Visual	Location	4.9903	0.0032	Manual
Ikea	Visual	Location	4.2969	0.0040	Manual
Train	Visual	Location	3.5485	0.0016	Manual
Wedding Venue	Visual	Location	3.1445	0.0008	Manual
Cafe	Visual	Location	3.1429	0.0056	Manual
Soccer Field	Visual	Location	2.6180	0.0016	Manual
Restaurant	Visual	Location	2.5640	0.0040	Manual
Record Store	Visual	Location	1.9834	0.0032	Manual
Punch Bar	Visual	Location	1.8196	0.0016	Manual
Elevator	Visual	Location	1.5206	0.0032	Manual
Car	Visual	Location	1.1889	0.0016	Manual
Bus	Visual	Location	1.0577	0.0040	Manual
Theater	Visual	Location	0.8183	0.0048	Manual
Gallery	Visual	Location	0.5972	0.0008	Manual
Family Home	Visual	Location	0.4279	0.0008	Manual
Beach	Visual	Location	0.3189	0.0008	Manual
Other Cafe	Visual	Location	0.2982	0.0008	Manual
Summer Child Bedroom	Visual	Location	0.2744	0.0008	Manual
Tom Child Bedroom	Visual	Location	0.1948	0.0008	Manual
Days of Summer	Visual	Transitions	2.1091	0.0270	Manual
Camera Cuts	Visual	Transitions	0.7619	0.7619	Automated
Scene Cuts	Visual	Transitions	0.1058	0.1058	Manual
**Total: 53**		**Total: 7**			

For each label, we provide the stimulus modality (Stimulus), label category (Category), and percentage of on-screen appearances (Appearance Percentage). We also include the Onset Percentage, defined as the percentage of label transitions from absent to present across the film (number of transition frames divided by the total number of frames). The method of annotation creation is given in the Method column. Manual: manually annotated using Advene and reviewed by two raters. Automated: created using segmentation software or pre-trained networks. Mix: created using a network fine-tuned on manually-created labels. *Note: “Indoor” and “Outdoor” are complementary categories and are consolidated into a single annotation titled “indoor-setting” within the dataset. In the dataset, a value of 99 is used to denote locations that are neither strictly defined as indoor nor outdoor such as a title card.

Movies as stimuli are characterized by various concurrent temporal scales and a dense range of simultaneous features. These characteristics make paradigms which use movies difficult to analyze using conventional methods designed for static and isolated presentations of images. However, recent developments in machine learning make it possible to identify and quantify relationships between these complex features and continuous neural activity^34^. As analytic tools and methods advance, this dataset can be used to address increasingly nuanced questions about human cognition. In particular, this dataset^26^ will be potentially relevant for researchers investigating natural scene and object representation and the multimodal integration of visual and auditory features. This dataset will also be of use to those investigating the structure and progression of narrative content, coding of emotions and arousal, predictive coding, and neural representations of interpersonal relationships.

## Methods

### Participants

We recorded from 29 patients with pharmacologically intractable epilepsy (age range, 19–62 years; median age, 40 years; 17 female patients; Table 1, Fig. 1b–d) while each underwent continuous monitoring to localize the seizure-onset zone through implanted depth electrodes. Patients with applicable implantation schemes were recruited through recommendation by the surgical staff based on interest in participating in single-unit studies. The study was approved by the Medical Institutional Review Board of the University of Bonn (accession number 095/10 for single-unit recordings in humans in general and 243/11 for the current paradigm) and adhered to the guidelines of the Declaration of Helsinki. Each patient gave informed written consent for the implantation of microwires and the recording and sharing of de-identified data, which included the right to drop out of the study at any time. We have taken the following steps to protect participant information: patients are identified in this dataset via a single numerical ID (Table 1); only the year of implantation is given; and no identifying information beyond gender and age at implantation are provided.

### Task and stimulus

Each participant watched a German-dubbed version of the film *500 Days of Summer* (2009) in its entirety (83 minutes). The movie was chosen as a stimulus because the cast was largely unknown to a general German audience at the time of the first recording sessions. Participants could pause the movie at any time and were instructed to do so if clinical interruptions occurred, such as measuring blood pressure. Of the 46 patients initially recorded, 17 were excluded because of major playback disruptions that precluded the reconstruction of a continuous viewing experience. We defined these disruptions as instances where frequent pauses or skips made it impossible to recover the movie playback in its original order, due to unwatched sections of the film. This left a final analytical sample of 29 patients who watched the film in its entirety.

The movie was presented in letterbox format on a laptop using a version of the open-source Linux package FFmpeg^35^ modified to output the Unix timestamp associated with each displayed movie frame to a log file. To synchronize between the movie presentation and the concurrent neural recordings, a separate script was run in the background of the movie presentation which sent transistor-transistor logic (TTL) pulses to the recording system every second using a data acquisition (DAQ) device connecting the presentation laptop to the data acquisition amplifier. This clocking script also logged the laptop’s system time immediately before and after each pulse. The arrival time of each TTL pulse was separately logged in an event file on the data-acquisition computer, creating a corresponding event timestamp in the neural recording system’s time coordinates. These timestamps were later used to define a linear transformation between the presentation timestamps of each frame in the movie to the neural recording system’s clock. The average transformation error between the two timescales was 2.30 ms (SD: 11.66 ms), providing an indication of the alignment between the stimulus presentation clock and the neural recording clock. Given the 40 ms frame resolution, this error is well below the duration of a given frame.

Pauses and skips in the movie playback were identified through the output of the modified FFmpeg program. For all included sessions, we processed the playback record by removing the segments corresponding to pauses and joining the moment before each pause directly to the moment after it. This transformed the watchlog into a complete, continuous representation of the movie which we used as a basis of exclusion for patients who had not watched the entire film or who had watched discontinuously by seeking within the playback. Across the 29 patients, the average number of pauses was 3.14 (SD: 2.22, median: 3). For the 91 total pauses recorded across all participants, the mean duration was 89.6 s (SD: 272.6 s, median 19.4 s). Although this procedure may have introduced minor noise due to abrupt transitions in neural activity, it facilitated the creation of a synchronized dataset across all participants and ensured a consistent movie stimulus despite the complexities introduced by the clinical setting.

Source code for the modified FFmpeg and clocking script are available in the GitHub code repository for this dataset^26^ under the directory paradigm_code^36^ (see Code Availability).

### Electrodes and data acquisition

Patients were implanted with depth electrodes (AdTech, Oak Creek, WI, USA) for the purpose of localizing the seizure onset zone for potential resection^37^. Microwire bundles (AdTech) were inserted through the hollow shaft of the depth electrode to record single neurons from implanted regions of interest. Each bundle consisted of eight insulated high-impedance wires and a single low-impedance reference wire, with an individual wire diameter of 40 *μ*m and common approximate distance of 4 mm from the tip of the depth electrode. The specific locations were chosen for implantation exclusively by clinical indicators, with an average of 9.97 (range: 4–12) microwire bundles implanted per patient (Fig. 1b).

Signals from the microwires were sampled at a rate of 32,768 Hz, band-pass filtered between 0.1 and 9,000 Hz, and amplified using a Neuralynx ATLAS Acquisition System with 256 channels (Neuralynx, Bozeman, MT, USA). The sessions for patients up to and including 16 were recorded using Cheetah (v.1.1.0, Neuralynx), all subsequent patient sessions were recorded using Pegasus (v.2.1.1 and v.2.2.0, Neuralynx). This dataset^26^ contains the spike trains and corresponding average waveform for each detected unit (see Data Availability).

### Electrode localization

Prior to implantation, the areas of interest were determined exclusively by clinical indication. After implantation, we localized electrodes for each patient using the open-source program LeGUI^38^ and co-registered pre-implantation structural MRI and post-implantation computed tomography (CT) images. To improve certainty in the estimated location, all microwire bundles were reviewed in the given patient’s native-space using the original co-registered volumes and manually classified by two separate raters with neurological training. An overview of the localized regions is given in Table 1 in the “Regions” column. Bundles on which the raters disagreed were further checked by an additional rater, and the channel was assigned the region with the greatest agreement. Due to the diameter of the microwires (0.04 mm) and the resolution of the co-registered scans, it was not possible to confidently locate each individual microwire. We therefore report the average x-, y-, and z-component of the bundle for each wire, such that all microwires in a bundle share a single set of Montreal Neurological Institute (MNI) coordinates.

### Spike sorting and data processing

Data were spike-sorted using Combinato^39^, which identifies putative neurons automatically through two stages of supraparamagnetic clustering. After automatic clustering, an experienced rater manually validated the results and categorized each putative neuron as either an artifact, multi-, or single unit considering the following criteria: 1) mean waveform, 2) inter-spike interval (ISI) distribution, 3) distance from the detection threshold, 4) rate of ISI violations (ratio of ISIs under 3 ms^40^), and 5) the distribution of spikes across the recording session. For the sake of simplicity, we refer to both single- and multi-units as neurons. After manual review, artificially duplicated spike events were removed automatically using our Duplicate Event Removal algorithm^41^.

**Fig. 6:**
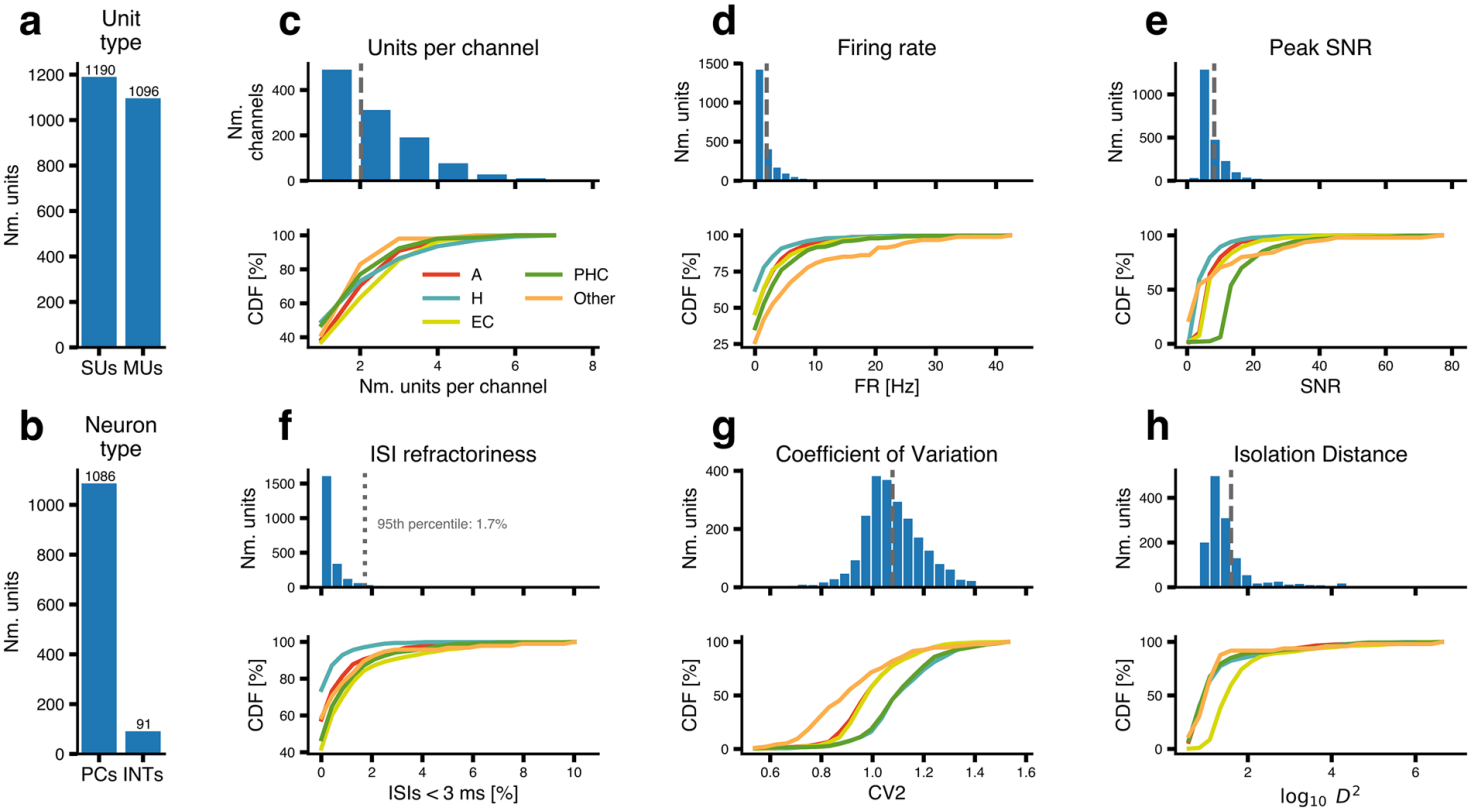
Neuron overview and spike sorting metrics. (**a**) We identified a total of 1,190 single units (SUs) and 1,096 multi-units (MUs) in the dataset after automatic spike sorting and manual review. (**b**) Single units in the dataset comprise 1,086 putative pyramidal cells (PCs), 91 putative interneurons (INTs), and 13 units with negative peaks (not pictured), classified according to spike width. (**c**) Number of neurons recorded per wire across all sessions, with an average of 2.02 ± 0.04 (mean ± s.e.m., dashed grey line). Only wires with at least one neuron are included. Cumulative distribution functions (CDF) are shown for each brain region separately in the lower panel. (**d**) Distribution of firing rates across all neurons (Hz) (1.93 ± 0.06 spikes/s, mean ± s.e.m.; dashed grey line). (**e**) Signal-to-noise ratio (SNR) of each neuron’s waveform peak (8.19 ± 0.10, mean ± s.e.m.; dashed grey line). (**f**) Percentage of inter-spike intervals (ISI) shorter than 3 ms (0.42% ± 0.01, mean ± s.e.m.). Over 95% of neurons exhibited less than 1.72% ISIs under 3 ms (dotted grey line). (**g**) Distribution of modified coefficient of variation values across all neurons (1.08 ± 0.003, mean ± s.e.m.). (**h**) Distribution of isolation distances with a median value of 21.82 for the 1,395 units for which this metric could be calculated.

Spike sorting quality metrics for the collected sessions are shown in Fig. 6. We assessed the sorting quality of each neuron using the following metrics: 1) peak signal-to-noise ratio (SNR), calculated as the maximum amplitude of the average waveform for all spikes of a given neuron over the median absolute deviation of the bandpassed (300 to 3000 Hz) continuous signal^42,43^ (Fig. 6e), 2) the proportion of ISIs under 3 ms^40,43,44^, 3) the robust coefficient of variation (CV2)^45^ (Fig. 6g), and 4) the isolation distance^46,47^ (Fig. 6h). To adapt the original isolation distance metric to the single-channel case, we defined a feature space using the top ten principal components calculated from the energy-normalized waveforms of all spikes and artifact events recorded from a single channel and the energy, defined as the average squared amplitude of a waveform. Isolation distances for a given cluster were calculated as Mahalanobis distances within the resulting 11-dimensional space^47^. In addition to the spike sorting metrics, we categorized single neurons as putative pyramidal cells or putative interneurons based on the peak-to-trough width of a given neuron’s spike shape^48,49^ using a threshold of 0.65 ms^50^ (Fig. 6b).

### Movie annotations

The stimulus consisted of natural scenes presented continuously and dynamically, providing a rich source of visual and auditory input. To organize the complexity of the stimulus, we generated high-resolution annotations of the movie, yielding a set of 53 labels on a frame-by-frame level (Fig. 2). These annotations were created either manually or through automated methods, using tools for detection and recognition. Manually created labels were developed through a collaborative consensus process. An initial set of annotations was created by one rater and subsequently refined by a second reviewer. All discrepancies were resolved through discussion until a unanimous consensus was reached; as such, we report final agreed-upon labels rather than inter-rater agreement metrics. The labels fall into seven categories: characters, character-related elements such as face appearance and general visibility, speech occurrences, locations, visual transitions and an interposed scene from another movie (*The Graduate*).

Throughout the movie, there are recurring frames featuring a drawing and a number indicating the specific day out of the 500 days covered in the storyline. These frames serve as time markers, introducing narrative jumps and reflecting a distinct visual pattern. Although the drawings are stylistically consistent, the images vary slightly depending on the depicted time of year. We assigned a dedicated label to these recurring frames and refer to them as Days of Summer.

Annotations for characters, character-related elements, locations, and the interposed scene were produced manually using the annotation software Advene^51^. Face labels for specific characters were obtained by fine-tuning a pre-trained neural network on facial images from the movie and generating labels based on the network’s predictions in conjunction with the general character labels. Character-specific face annotations were generated via a two-stage training pipeline using MTCNN (Multitask Cascaded Convolutional Network)^52^ for face detection and InceptionResnetV1 (pretrained on VGGFace2^52^) for embedding extraction. We first trained a linear face classifier network in a supervised manner on a curated subset of 200 images per character, then refined it through a weakly supervised stage on 80,000 frames where face crops were matched to frame-level character presence labels using the Hungarian algorithm. The final dataset was produced by applying the classifier to the full movie, followed by a post-processing filter to remove any face predictions that contradicted established ground-truth character presence. Prior to post-processing, the classifier achieved 93.94% accuracy on the held-out test set (the final 40,000 frames of the movie), where a prediction was defined as correct if the identified faces were a subset of the general character labels. Although this metric serves as a proxy due to the lack of face-level annotations, the subsequent post-processing stage ensures that all final positive face labels are a strict subset of validated character presence, effectively eliminating false-positive character assignments. Manual annotation was used to label the voices of the two main characters, as well as the segments for Days of Summer and all Scene Cuts. Camera Cuts, on the other hand, were detected automatically using the open-source algorithm, PySceneDetect^53^, run with default parameters (adaptive detection, threshold: 3, frame window: 2, minimum content value: 15, weights: 1) and manually reviewed. Both Scene and Camera Cuts are categorized as visual transitions defining the event structure of the dynamic stimulus. For these annotations, transitions were annotated by assigning a positive label (1) to the first frame of each new segment. All annotations are provided as binary labels, indicating presence or absence (see Fig. 3 for an example set of frames). The Indoor/Outdoor label is the sole exception: it functions as a categorical variable where 1 represents indoor locations and 0 represents outdoor locations. In instances where a frame cannot be strictly classified as either indoor or outdoor, such as a title card or credit sequence, the label is assigned a value of 99.

## Data Records

All session data included in the dataset^26^ are provided in the Neurodata Without Borders (NWB) format^54,55^ and were compiled using the PyNWB Python package^56^. We generated a separate and standalone NWB file for each movie viewing session - corresponding to one file per patient - such that all electrophysiological data, movie annotations, and synchronization files necessary for analysis are included in a single file. The general structure of a session file is given in Fig. 4, and specific descriptions for the contents and organization of each type of data are given below. The NWB files are available as a DANDI dataset (10.48324/dandi.001616/0.260702.0824).

The scripts used to generate the NWB files are given in the associated GitHub repository, alongside all code used for figure creation^36^ (see Code Availability).

### Single-neuron data

Each NWB file includes the spiking data and the electrode meta-data. All spiking data are stored in the \units container and are structured as a table in which each row represents an individual neuron and each column represents a separate item of information. For each neuron, we provide the spike times in milliseconds aligned to movie stimulus onset (\units[spike_times]) as well as the mean and standard error of the mean of the waveforms (\units[waveform_mean, waveform_sem]). Each waveform contains 64 samples of bandpass-filtered signal in microvolts, with the peak spike amplitude positioned at the 20th sample. The \units table also contains the spike-sorting metrics for each neuron (\units[peak_SNR, isi_violations, cv2]), as well as an indicator denoting whether the neuron was labeled as a single (\units[is_single_unit] == 1) or a multi-unit ( == 0) and the neuron’s cell type (\units[cell_type], either putative pyramidal cell or putative interneuron). We also include each neuron’s hemisphere (\units[hemisphere]), region (\units[brain_region]), and the continuously sampled channel (CSC) number that links the neuron to its corresponding microwire (\units[csc_nr]).

Electrode localizations are organized using two containers, \electrode_groups and \electrodes. Device details and depth electrode locations are provided in \electrode_groups. Information about individual channels is provided in \electrodes as a table, where each row represents a channel and each column specifies a channel property. We only include channels which contained neurons, and all neurons recorded from a given channel can be indexed by the csc_nr value. Each channel included in the \electrodes table inherits its group and group name from \electrode_groups according to its corresponding depth electrode. As noted in the Methods, the final locations of implanted microwires may differ from the clinical electrode locations. We include the expert-assigned location as \electrodes[brain_region]. For completion, the originally planned locations are included in the descriptions of the \electrode_groups entries, although only the expert-assigned location should be considered for analysis. Hemisphere is denoted in \electrodes[hemisphere] and the MNI-coordinates for each channel are provided by the \electrodes[x, y, z] columns. Finally, the specific microwire index number is given in the column \electrodes[bundle_index].

### Movie stimulus

Each patient watched a German-dubbed version of the film *500 Days of Summer* in its entirety and without subtitles. Due to copyright protections, we cannot include the film stimulus in the public release of the dataset. To enable others to add to the set of annotations and to investigate frame-wise content, we offer a set of look-up tables and a code module that allow users to align our experimental paradigm with an official version of the movie (Version: Cine Project (2010 Release), EAN: 4010232049162, ASIN: B0030FXXLK). This ensures that users can map our findings and annotations onto a standard reference copy. These tables, along with scripts for creating our version and for obtaining frames, are available in the code repository^36^ (see Code Availability).

The movie used in this study has a frame rate of 25 frames per second (fps), meaning each frame was displayed for 40 ms. The full movie has a duration of 5029.68 s, corresponding to a total of 125,743 frames. In our previous work, we excluded the beginning and end credit sequences and analyzed the section from 36.72 s to 4763.2 s (118,162 total frames)^34^. Each NWB file includes the presentation timestamps and corresponding frame indices (\stimulus[movie_frame_times_base]). To coordinate with frame onsets, binned spike times should use multiples of the frame duration as window lengths and should align with presentation timestamps (pts). Depending on the specific research question, different bin lengths may be required to capture distinct neural dynamics. Shorter bins (40–80 ms) resolve rapid, stimulus-locked dynamics and fine-grained timing, while longer bins (200–480 ms) aggregate the higher spike counts needed for stable population decoding or identifying slower state transitions. To support flexible analysis, we provide a reference table in \processing[machine_learning][movie_binning_info] containing movie bin edges for bin lengths of 40 ms, 80 ms, 200 ms and 480 ms. For each bin length, the table includes bin edges both in pts (column: edges) and as movie frame indices (column: frames).

### Viewing sessions

The movie was aligned with the neural data using a clocking script that sent a TTL pulse to the data acquisition system every second, as described in the Methods (see Task and stimulus). For completeness, the NWB file includes the original synchronization logs for each movie session in the NWB file \stimulus[raw_watchlogs], although all movie annotations and related information have been converted to the neural time coordinates. To facilitate population analyses, we also provide cleaned synchronization logs under \stimulus[cleaned_watchlogs]. These were preprocessed to remove any skips or pauses; while all patients watched the movie in its entirety - frame-by-frame - any sections where the movie was paused have been excluded. This allows for the straightforward concatenation of frame-by-frame data across the whole population.

### Movie annotations

We provide frame-wise labels indicating the presence of 53 features in the film (Table 2) in each NWB file under the table \stimulus\annotations_base. Annotations are provided as a set of binary labels where the value 1 indicates presence and 0 absence. The movie-aligned annotations are available in a format based on onset and offset times, marking intervals of occurrence or absence. Two arrays of timestamps define consecutive segments of the movie (indexed by array position), specifying where a feature is present or absent. The columns start_time and stop_time store the onset and offset times (in pts timestamps). The columns label_name and entry_index identify the feature and the segment index, while value indicates whether the feature occurs (1) or not (0). Note that the number of segments is smaller than the total number of frames, as consecutive frames with identical labels are grouped into single segments.

Indicator functions for the German edition of *500 Days of Summer* are available under \processing[machine_learning][movie_annotations_indicator_functions]; these are aligned with both the base movie version and the cleaned watchlogs for each patient. In contrast to the base annotations, the indicator function aligns with the number of frames in the stimulus. Note that the movie-aligned annotations and indicator functions are the same in every NWB file. Each annotation was additionally aligned to the raw watchlog of each patient, such that any pauses or alterations in playback are directly reflected in the annotation sequence. These aligned annotations are included in the corresponding NWB file under the table \stimulus\ annotations_patient_aligned.

### Data splits

We emphasize the need to carefully split the data into training, test, and validation sets when using the data in a machine-learning pipeline. To account for the high temporal autocorrelation inherent in both the neuronal data and movie stimuli, we provide a dedicated splitting mechanism. Without such partitioning, the high similarity between adjacent samples leads to data leakage, which inflates predictive performance and obscures the neural representations under study.

We address this by segmenting the data and introducing temporal buffers between the training, validation, and test sets. Specifically, we employ a 5-fold cross-validation scheme incorporating these buffers to mitigate data leakage and ensure representative performance metrics (Fig. 5a).

Our evaluation on movie-feature prediction shows that while a lack of buffers produces over-optimistic results, increasing the buffer length reveals the true, lower baseline of predictive performance (Fig. 5b). Consequently, we recommend a 32 s buffer to ensure independence between sets. The splitting mechanism is available in the associated GitHub repository (see Code Availability, ML_framework\dataloader.py), and a comprehensive discussion of buffer effects is provided in our previous work^34^.

## Technical Validation

### Single-neuron data and spike-sorting quality metrics

We recorded 2,286 neurons from 29 patients while each watched the movie *500 Days of Summer* in its entirety. On average, each patient received approximately 10 Behnke-Fried electrodes (Table 1) and each session yielded an average of 78.83 ± 5.44 neurons (mean ± s.e.m., Fig. 1d). Considering only microwires with valid units, we found an average of 2.02 ± 0.04 neurons per wire (mean ± s.e.m., Fig. 6c) and an average firing rate of 1.93 ± 0.06 Hz (mean ± s.e.m., Fig. 6d). We used several metrics to assess the quality of the spike-sorted recordings. For each neuron, we calculated the following: (1) peak signal-to-noise ratio (SNR), calculated as the maximum amplitude of the average waveform for all spikes of a given neuron over the median absolute deviation of the bandpassed continuous signal (300 to 3000 Hz)^42,43^, with an average of 8.19 ± 0.10 (mean ± s.e.m.) for all cells (Figs. 6e), (2) the percentage of inter-spike intervals (ISIs) under 3 ms (absolute refractory period)^43,44^, all neurons: 0.42% ± 0.01% (mean ± s.e.m.); Fig. 6f), (3) the robust coefficient of variation (CV2)^45^ (1.08 ± 0.003, mean ± s.e.m.; Fig. 6g), and (4) the isolation distance^46,47^ (median value of 21.82, Fig. 6h). Our isolation distance metric is an extension of Schmitzer-Torbert *et al*.^47^ to the microwire case and requires that the number of spikes in a given unit be less than the total number of spikes detected on the unit’s channel. For channels with an active multi-unit and relatively few single-units, the chance that the multi-unit violates this constraint is high.

Each neuron was manually categorized as a multi- or single-unit based on commonly used criteria (see Methods) by an experienced rater. Across all sessions, we identified 1,190 single-units and 1,096 multi-units (Fig. 6a). Within the single-units, we identified 1,086 putative pyramidal cells, 91 putative interneurons (Fig. 6b), and 13 neurons with negative peaks for which we did not assign putative cell types. All described values are included in the NWB files as columns in the \units container.

### Responses of individual neurons to movie features

**Fig. 7:**
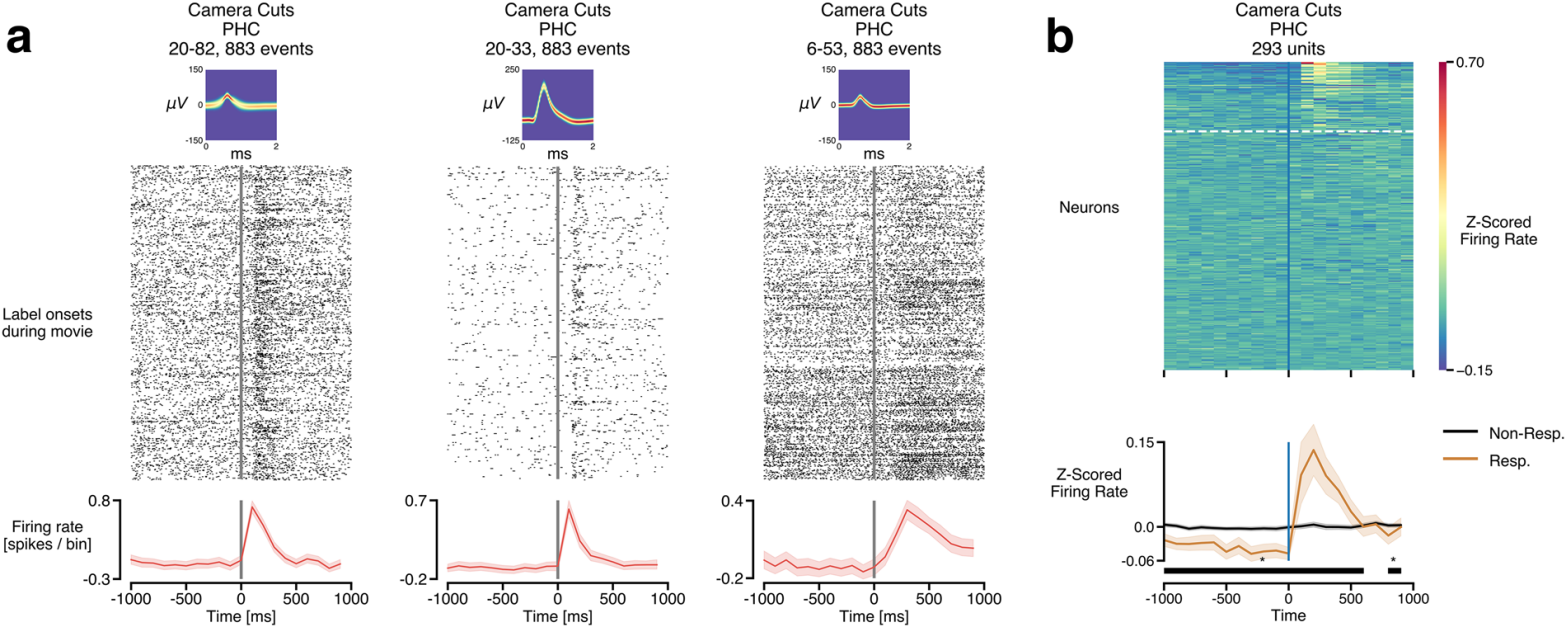
Example neuron responses to the movie feature Camera Cuts. (**a**) Individual neurons with significant responses to the movie feature Camera Cuts (ZETA test^57^, *p* ≤ 0.01, 1 s post-stimulus window, 1000 permutations). Top panel: waveform mean and standard error. Middle panel: spiking activity following Camera Cut events. Onset events were filtered to remove events that occurred less than 1000 ms after a previous cut. Bottom panel: Spikes per 100 ms surrounding the Camera Cuts, averaged across all valid events in the movie. Solid line denotes average spiking activity; shaded area denotes 95% confidence interval. (**b**) Responses to Camera Cut events across all parahippocampal neurons. Top panel: Activity of each parahippocampal neuron surrounding valid Camera Cut events (1000 ms before and after the Camera Cut event at 0 ms, blue line). Each row depicts the bin-wise spiking activity of an individual neuron averaged across all Camera Cut events in the movie. White dashed line separates significantly responsive neurons (above the line) from non-responsive neurons (below). Bottom panel: Time course of the responsive neurons (orange line) versus the non-responsive neurons (grey line). Black horizontal inset bars indicate periods of significant difference between the responsive vs. non-responsive curves (cluster permutation test, *p* ≤ 0.05). Adapted from Gerken, Darcher, *et al*.^34^.

To validate the presence of single neurons tuned to movie features in the dataset, we report the activity of individually responsive neurons to their preferred movie feature. We define a neuron as responsive to a given movie feature if the stimulus-aligned spiking significantly deviates from a linear cumulative distribution function during the post-stimulus window (Zenith of Event-based Time-locked Anomalies, ZETA^57^, *α* = 0.01, 1 s post-stimulus window, 1,000 permutations). Each appearance of the labeled feature in the movie marked the onset of a ‘trial’. Trials for which the tested feature appeared less than a full second prior to onset (e.g. Summer was off-screen for less than a second or a Camera Cut occurred less than a second before the next cut event) were excluded from analysis. Example neuronal responses for the Camera Cuts events are shown in Fig. 7a. Of the annotated movie features, we found the greatest number of neuronal responses to the Camera Cuts (195/2,286 neurons; ZETA, *p* < 0.01) and to the Scene Cuts (93/2,286; ZETA, *p* < 0.01). The parahippocampal cortex contained the largest proportion of neurons with significant responses to the visual transitions (Camera Cuts, 68/293 neurons, see Fig. 7b; Scene Cuts, 32/293 neurons). We additionally identified time points during which the firing activity of responsive neurons differed from non-responsive neurons using a cluster permutation test^58^. As shown in Fig. 7b, the firing rate of the identified responses significantly increased after Camera Cut events compared to the firing rates of non-responsive parahippocampal neurons (cluster permutation test, *p* < 0.001). We found comparatively fewer responsive neurons for character-related features, most of which were identified in the amygdala (Tom: 45/2,286 (19 amygdala); Summer: 38/2,286 (17); McKenzie: 7/2,286 (2); Persons: 32/2,286 (12); ZETA, *p* < 0.01). For this validation analysis, we tested seven movie features (Tom, Summer, McKenzie, Persons, Scene Cuts, Camera Cuts, and Indoor/Outdoor), and expected approximately 155 spuriously responsive neurons across the tested population (FWER: 6.79%), with approximately 23 false positives expected within each feature test independently.

### Decoding from populations of neurons

**Fig. 8:**
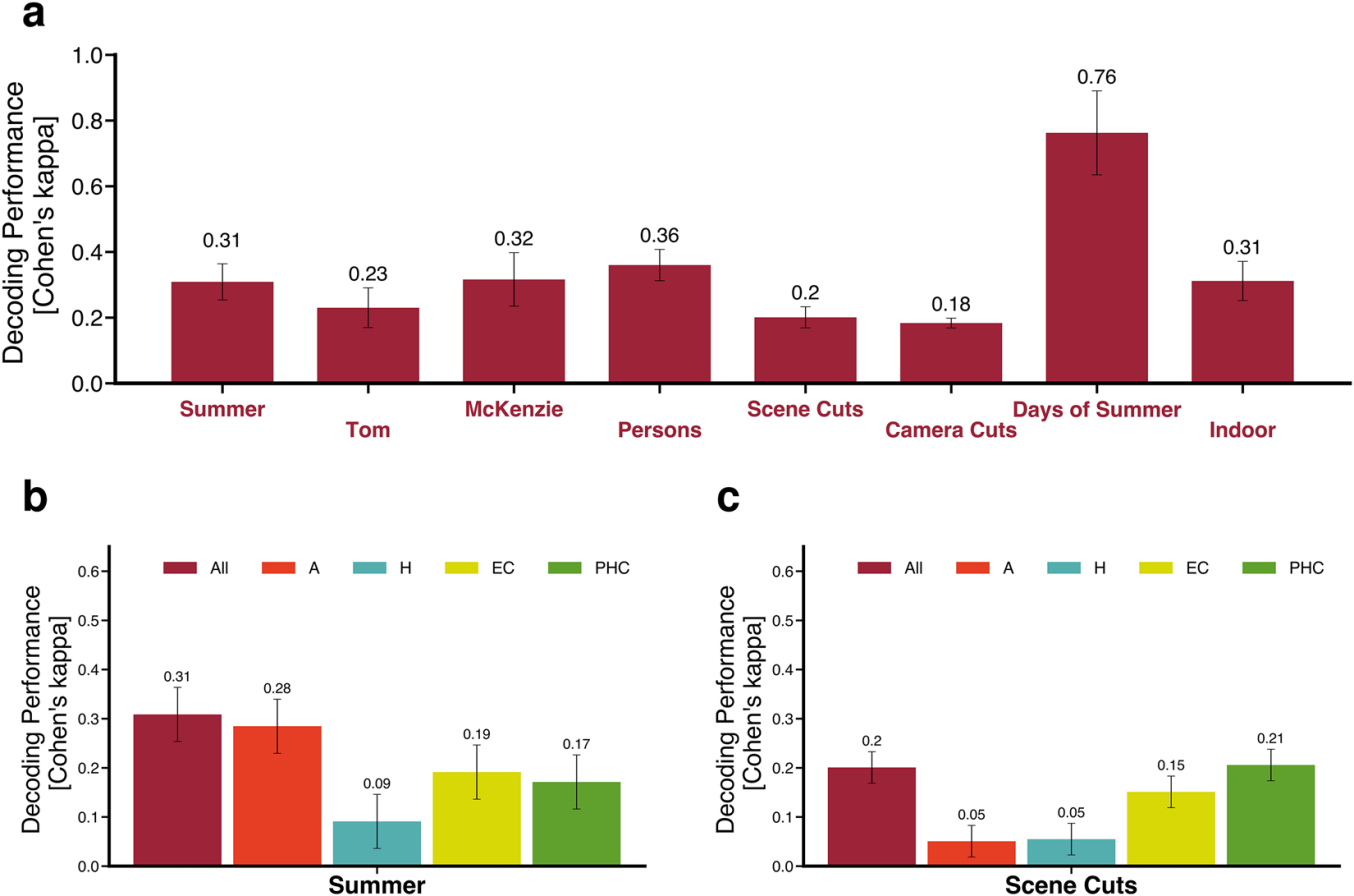
Decoding performances from a pseudo-population of neurons pooled across patients. (**a**) Decoding performance for the three main characters of the movie, indoor settings, and visual transitions was measured using Cohen’s kappa, where a score of 0 represents chance and 1 represents perfect classification. (**b**) Among MTL subregions, the amygdala achieved the highest decoding accuracy for the character Summer, with a performance comparable to that of the entire MTL population. (**c**) For the Scene Cuts label, the parahippocampal cortex was the top-performing subregion.

The relative absence of individual neuronal responses to many of the annotated movie features may suggest that information is either represented only at the population level, or that the movie’s inherent dynamic nature precludes detection using traditional approaches. In our previous work^34^, we investigate the population representation by decoding the presence of the main characters, indoor setting, and visual transitions (Fig. 8a) on a frame-by-frame basis from a pseudo-population of neurons pooled across patients. For these analyses, we selected the annotated features with the highest consistent presence throughout the film (Table 2) and trained a Long Short-Term Memory-based model for each feature. In all cases, decoding performance significantly exceeded chance levels, as measured by Cohen’s kappa weighted against a shuffled test set. We also found that decoding performance varied significantly by brain region. The amygdala was the top-performing region for identifying characters. For instance, when decoding the character Summer, the amygdala achieved the highest score across all regions (0.28 Cohen’s kappa, Fig. 8b).

In contrast, the parahippocampal cortex was most effective at decoding visual transitions (0.21 Cohen’s kappa for Scene Cuts, Fig. 8c). These results demonstrate that the population of neurons contains information relevant to the movie features, and that the contents of a given frame can be decoded from the pooled population activity.

## Usage Notes

To facilitate use and reproducibility, we provide a comprehensive Python-based ecosystem via our GitHub repository^36^ containing processing modules, analysis pipelines, and a set of instructive Jupyter notebooks. We recommend that users follow the Quick Start Guide provided in the repository’s README, which includes instructions on how to set up an appropriate Python environment, how to download the data, and how to work with the codebase. For general exploration, users can utilize the provided visualization notebooks to inspect NWB files directly from the DANDI archive (DANDI:001616)^26^. For analyses involving unit-specific response properties, such as latency, we suggest that users restrict their neuronal set to only putative single units. For group-level analyses, we encourage users to pool neurons across individual patients and unit type into a pseudo-population, since the data are precisely aligned across sessions and individual recordings. This approach facilitates population-level encoding and decoding by leveraging the collective coverage of specific brain regions across the cohort.

The existing 53 labels can be directly applied to individual neurons or patient recordings, or to a pseudo-population, for statistical analysis or decoding approaches. To facilitate users who want to apply an encoding approach or create additional labels, we have included a wrapper utility that recreates the version of the movie shown to patients from publicly available versions of the movie. This tool provides a direct mapping between the original 125,743 frames and their equivalents in DVD releases, accounting for differences in frame layout or skipped frames. To support deeper inspection of the visual stimuli, the wrapper utility also includes a frame-export function to convert the movie into individual image files, as well as a visualization tool to inspect the stimulus alongside the experimental metadata stored in the NWB files.

For machine learning applications, we provide a PyTorch Lightning framework with a pre-configured 5-fold cross-validation splitting strategy (see Data splits). This structure is specifically designed to account for the continuous nature of the stimulus; it utilizes a 32 s buffer between training and validation folds to mitigate temporal autocorrelation and prevent information leakage. We highly recommend that users apply this structure when designing a machine learning pipeline for analyzing this dataset^26^. We additionally suggest a default bin size of 80 ms for predictive tasks to balance temporal resolution with statistical power. Detailed instructions for handling task pauses and configuring local data paths are maintained in the repository’s Quick Start guide, and detailed instructions for setting up a machine learning pipeline are included in the corresponding section of the repository.

## Data Availability

The complete dataset, encompassing both neural data and stimulus data, is formatted as Neurodata Without Borders (NWB) files. The NWB-formatted dataset is available on the DANDI Archive: 10.48324/dandi.001616/0.260702.0824^26^. We are not able to directly release the movie stimulus used in this experiment. To facilitate access, we have created a set of Python-based modules (see following Code Availability section) that convert a specific DVD version of the movie, *500 Days of Summer*, to the version used in our experiment–DVD: Cine Project (2010 Release), EAN: 4010232049162, ASIN: B0030FXXLK.

## Code availability

All code used to generate NWB files, perform technical validation, synchronize movie stimulus versions, and visualize results is provided in a GitHub repository, https://github.com/mormannlab/SUMMER^36^. The calculation of the median absolute deviation (normalization factor for obtaining the peak signal-to-noise ratio of a channel^43^) is implemented in MATLAB. All other code is implemented in Python. We provide a conda environment file containing all used dependencies, as well as instructions for creating and using an environment. The previously published work on this data^34^ was performed using a relational database built for this dataset^59^.

Beyond traditional analytical workflows, we prioritize accessibility for the machine learning community and offer the dataset in an ML-ready format. To further lower the barrier to entry, our repository includes several modules for training a simple neural network on two demonstrative tasks. Detailed setup instructions are provided to ensure ease of use and facilitate rapid implementation.
